# An empirical analysis of double reduction education policy based on public psychology

**DOI:** 10.3389/fpsyg.2022.952719

**Published:** 2022-08-30

**Authors:** Xin Zhang, Weibin Zhao, Kai Zhou

**Affiliations:** ^1^School of Foreign Languages, Changchun University, Changchun, China; ^2^School of Cyber Security, Changchun University, Changchun, China

**Keywords:** double reduction policy, educational equity, difference analysis, structural model equation, educational psychological cognition

## Abstract

To effectively reduce the homework burden and off-campus training burden of students in the compulsory education stage in China, China adopts the double reduction policy to carry out corresponding governance in the field of education. This study aims to explore the public's psychological cognition of the current education system under the implementation of the double reduction policy. The public's opinion data on education concepts under the implementation of the double reduction policy are collected, then the SPSS software and Amos software to quantitatively analyze the public's educational concept data are used, and a corresponding structural model equation to analyze the public's opinion on the implementation of the double reduction policy is established. The psychological concepts of all aspects of education are observed, the public's psychological views on education under the implementation of the double reduction policy are finally obtained, the ideological connotation of popularizing the double reduction policy for the general public is provided, and the balanced development of the domestic education system for the implementation of the double reduction policy is ensured. Two suggestions are made in order to provide a corresponding reference for the public's psychological cognition research on the current education system under the implementation of the double reduction policy.

## Introduction

In July 2021, China promulgated the “Double Reduction Policy”, which was deployed to solve the problem of excessive homework burden and off-campus training burden on students in China. It aims to prevent China's education system from being invaded by capital, monopolize China's education resources, and then subvert China's education system. To this end, China has implemented the “double reduction policy” (Zhang, 2021) to reconstruct and manage the current public education system, remove the capitalization of the current education system, ensure the education fairness of the Chinese people, and rebuild the public's understanding of public education. The educational psychology of the system.

To explore the psychological cognition of the public about the current education system under the implementation of the double reduction policy in the field of education in China, this study first uses a questionnaire to investigate the public's educational resources, on-campus teaching quality, and education under the implementation of the double reduction policy. The training direction, the governance of off-campus training, and the opinion data of China's public education ecosystem are collected, and the public's educational psychological concept under the implementation of the double reduction policy is quantified through the structural model equation, which has been implemented in China's education field. After the double reduction policy, the public's psychological cognition toward the education system, and finally, according to the public's psychological cognition toward the education system, corresponding substantive suggestions for the implementation of the double reduction policy are put forward, so as to provide empirical evidence for the subsequent double reduction policy. The research provides a reference to the corresponding academic achievements.

## Literature review of education ideas under double reduction policy

### The reform of educational psychology under the double reduction policy

The “double reduction policy” reforms the education system in and out of school in order to lead the whole society to change the psychological concept of education, promote schools to play the main function of teaching and educating, gradually manage the training courses of off-campus education and training institutions, comprehensively improve the teaching quality of the school, change the educational psychology of the public, and cultivate talents with all-round development of virtue, intelligence, physical conditions, mind status, and community service (Ma et al., 2021), so as to win the psychological recognition of on-campus education from the public. Under the implementation of the “double reduction policy,” the current public education psychology can be changed to a certain extent.

### The psychological transformation of family education under the double reduction policy

At present, families pay special attention to their children's education investment. In addition to the daily education expenditures in school, many families will also provide tutoring for their children in off-campus courses, which greatly increases the family's investment in children's education, and will cause a certain economic burden to many families. Their children were allowed to obtain better educational resources without restraint in the competition of educational capital investment, which will eventually lead to a polarized situation in the allocation of educational resources.

By using the method of instrumental variables to study the psychology of family education under China's double reduction policy (Liu, 2021; Huang et al., 2022), China's education field compensates for the educational resources of disadvantaged families to a certain extent by implementing the “double reduction policy.” At the same time, it will change the psychological concept of family education in today's society to a certain extent, so that it will not make an excessive capital investment in children's off-campus education, and finally promote the development of China's public education system in the direction of diversification. It provides a strong guarantee for talents with all-round development of moral, intellectual, physical, and beauty.

## The research method of social mass education psychology under double reduction policy

### Selection of subjects

We used the random sampling method, and the respondents about the educational psychological cognition of the “double reduction policy” were selected from the urban area of Changchun, Jilin Province. The survey method adopted the form of a network questionnaire, and the respondents uniformly filled in the questionnaire on the questionnaire star platform. In this study, 350 questionnaires were sent out this time, and 305 valid questionnaires were collected in total, with an effective rate of 86.14%.

### Specific research tools

The questionnaire designed by scale was chosen as the research tool in this study, which included five dimensions of the equitable distribution of educational resources under the double reduction policy, the guarantee of in-school teaching quality, the diversification of national education and training directions, the impact of off-campus training on students, and the public education ecosystem in China.

In addition, on the premise of the implementation of the “double reduction policy,” the educational psychology of the public is taken as the research target, and the cognitive status of the public's educational psychology under the implementation of the “double reduction policy” is collected through the scale of the above five dimensions, so as to conduct research and analysis on the educational psychology of the public under the “double reduction policy.”

#### Educational psychological cognition scale of the public under the “double reduction policy”

This study adopts the Likert scale developed by Likert (Liu, 2021), an American social psychologist, to investigate the public's educational psychological cognition under the “double reduction policy.” The scale is mainly composed of a set of statements, each set of statements including “very dissatisfied,” “not satisfied,” “relatively dissatisfied,” “fair,” “relatively satisfied,” “satisfied,” and “very satisfied” seven kinds of assessment index, and uses the magnitude seven scoring system, from 1 (very dissatisfied) to 7 (very satisfied) to score of seven kinds of evaluation indexes. In this way, quantitative analysis is used to analyze the psychological cognition of social mass education under the implementation of the “double reduction policy.”

The psychological cognition scale of social mass education under the “double reduction policy” was compiled based on the Likert scale. The content of the scale of equitable distribution of educational resources and the impact of the educational ecosystem under the “double reduction policy” was based on Yu's (2021) Study on Ensuring Educational Equity by the Double Reduction Policy (Du, 2021). The content of the scale of teaching quality assurance in schools under the “double reduction policy” refers to Chen (2021)'s research on the promotion of classroom teaching quality by the double reduction policy (Zhao et al., 2020), “double reduction policy” under the direction of national education to cultivate diverse scale content is the reference to Ou and Xue (2021) about “Double Reduction Policy” in the Research of the Development of the Education Training Mode Diversification (Yu, 2021). The scale content of out-of-school training governance under the double reduction policy refers to Liu Junyan's Study on the Motivation of Family Out-of-School Training Demand and its Enlightenment to the Double Reduction Policy (Chen, 2021).

### Analysis methods and data processing steps

First, the data collected in the questionnaire were statistically analyzed, and then the corresponding data were analyzed. Finally, the results of the data were studied to obtain the public's psychology of educational cognition under the “double reduction policy.”

#### Analysis of non-dimensional data in the scale

First, demographic variables in the questionnaire were statistically analyzed, followed by a descriptive analysis of the demographic information in the questionnaire, and finally the characteristics of demographic information participating in the questionnaire were obtained.

#### Analysis of dimension data in the scale

First, reliability analysis was conducted on the data of each dimension in the questionnaire scale, and then the difference test was conducted on the data of each dimension based on demographic variable information to obtain different people's educational cognition views under the double reduction policy. Then, exploratory factor analysis and confirmatory factor analysis were conducted for validity. Finally, the corresponding structural equation model is established to analyze the public's educational cognitive psychology under the implementation of the “double reduction policy.”

## Analysis and research on the data model related to the double reduction policy

### Analysis of non-dimensional data in the scale

#### Demographic variable frequency statistical analysis of non-dimensional data in the questionnaire

From the 305 valid questionnaires collected, it can be seen that the demographic characteristics of the public's psychological cognition of the current education system under the implementation of the double reduction policy are shown in Table 1. In addition, by using the SPSS23 software to conduct a frequency analysis of demographic variables on the data in the questionnaire, we can see that, according to the frequency analysis of demographic variables mentioned above, the mean values of gender, age, identity, education background, and understanding degree of double reduction policy in this questionnaire are all close to the median value of the survey variables, i.e., gender is biased to female, age is biased to 29–39 years, identity is biased to parents, education is biased to junior college and bachelor's degree, and knowledge of the double reduction policy is biased to people who have a certain understanding of the double reduction policy (Zhang and Gao, 2021).

**Table 1:** Frequency analysis of demographic variables.

**Variable**	**Option**	**Frequency**	**Percentage**	**Average**	**Standard deviation**
Gender	Male	130	42.60%	1.57	0.5
	Female	175	57.40%		
Age	Below 18	17	5.60%	2.87	1.1
	18–28	125	41.00%		
	29–39	74	24.30%		
	40–50	59	19.30%		
	Over 50	30	9.80%		
Identity	Teacher	61	20.00%	2.58	1.08
	Student	85	27.90%		
	Parents of students	81	26.60%		
	Others	78	25.60%		
Education background	Below junior high school	27	8.90%	2.89	0.88
	Senior high school	54	17.70%		
	Junior college undergraduate	149	48.90%		
	Master degree or above	75	24.60%		
Understanding degree of the double reduction policy	Never heard of it	28	9.20%	2.65	0.81
	Only heard of the term	88	28.90%		
	Have some understanding of it	151	49.50%		
	Know very well	38	12.50%		

The standard deviations of gender, age, identity, educational background, and the understanding degree of the double reduction policy range from 0.5 to 1.1, indicating that the fluctuation range of demographic variables in the questionnaire is within the reasonable range of the data of demographic variables.

### Analysis of dimensional data in the scale

#### Reliability analysis of dimensional data in the questionnaire

The SPSS software was used to conduct a reliability analysis on the questionnaire data of the public's views on the current education system under the implementation of the double reduction policy, and the data results in Tables 2–7 were obtained. According to the reliability analysis in Tables 2–6, under double reduction policy education can view the fair distribution of resources, the school teaching quality can ensure views, about national education can develop the direction of diversification development view, and training outside governance in favor of students' learning and on double reduction policies can ensure the opinions about the development of China's public education ecological system on the five dimensions, the overall standardized coefficients are 0.880, 0.888, 0.851, 0.834, and 0.859 respectively, and the value of the reliability coefficient ranges from 0 to 1. The closer the reliability coefficient is to 1, the higher the reliability will be. Therefore, the reliability of each dimension is relatively good (Yu and Ao, 2022). According to the Cronbach's reliability coefficient after deletion of each dimension item, it can be seen that they are all less than the overall standardization coefficient. Therefore, there is no need to adjust the views of each dimension under the double reduction policy.

**Table 2:** Views on whether educational resources can be fairly distributed under the double reduction policy.

**Option**	**The mean scale value after deleting the item**	**The scale variance after deleting the item**	**Corrected term and total correlation**	**Square multiple correlation**	**Clone Bach Alpha after deleting the item**	**Standard α**
The overall expectation degree of the school and the government to implement the policy of reducing students' learning tasks	14.741	14.936	0.695	0.489	0.864	0.88
The degree of satisfaction with the distribution of educational resources in current living areas	14.941	13.187	0.776	0.61	0.832	
The overall degree to which local schools and governments are expected to ensure and promote educational equity	14.813	14.139	0.741	0.55	0.846	
Views on the implementation of the double reduction policy to ensure that the educational resources used by Chinese citizens achieve fair distribution	14.895	13.318	0.755	0.586	0.841	

**Table 3:** Views on whether in-school teaching quality can be guaranteed under the double reduction policy.

**Option**	**The mean scale value of the deleted item**	**The scale variance after deleting the item**	**Corrected term and total correlation**	**Square multiple correlation**	**Clone bach alpha after deleting the item**	**Standard α**
To the local school about education quality information understanding status	15.292	13.043	0.745	0.559	0.859	0.888
Views on whether the current normal school curriculum education can meet the needs of students in education	15.17	12.984	0.772	0.615	0.848	
Satisfaction degree with the local government's efforts to address the quality of school education	15.236	14.049	0.732	0.541	0.863	
The expected degree of improvement in your school's teaching quality under the double reduction policy	15.039	13.887	0.769	0.609	0.85	

**Table 4:** Views on whether national education and training direction can be diversified under the double reduction policy.

**Option**	**The mean scale value of the deleted item**	**The scale variance after deleting the item**	**Corrected term and total correlation**	**Square multiple correlation**	**Clone bach alpha after deleting the item**	**Standard α**
The overall expectation degree of Internet education and vocational education	10.361	5.448	0.754	0.575	0.754	0.851
The overall satisfaction degree of current Internet education and vocational education in China	10.328	4.932	0.686	0.475	0.824	
Overall satisfaction with the diversified development of national education and training direction under the double reduction policy	10.308	5.53	0.717	0.531	0.787	

**Table 5:** Views on whether the governance of off-campus training under the double reduction policy is beneficial to students' learning.

**Option**	**The mean scale value of the deleted item**	**The scale variance after deleting the item**	**Corrected term and total correlation**	**Square multiple correlation**	**Clone bach alpha after deleting the item**	**Standard α**
Opinions on whether training outside school is helpful to students' academic performance	10.138	5.534	0.7	0.489	0.76	0.834
Opinions on whether after-school service organized by campus can meet students' learning needs under the double reduction policy	10.187	5.422	0.703	0.494	0.758	
The overall satisfaction degree of after-school service implementation effect and quality organized by local schools	10.154	6.453	0.684	0.467	0.782	

**Table 6:** Views on whether the double reduction policy can ensure the development of China's public education ecosystem.

**Option**	**The mean scale value of the deleted item**	**The scale variance after deleting the item**	**Corrected term and total correlation**	**Square multiple correlation**	**Clone bach alpha after deleting the item**	**Standard α**
Opinions on whether the implementation of “double minus” policy can change the views of Chinese students' academic anxiety	10.564	5.931	0.723	0.554	0.814	0.859
Opinions on whether the implementation of “double reduction policy” is helpful to restrain utilitarian psychology in the ecological system of public education in China	10.502	5.731	0.799	0.64	0.74	
Opinions on whether the implementation of “double reduction policy” is helpful to the construction of China's public education system	10.495	6.593	0.684	0.487	0.847	

**Table 7:** Reliability coefficient of overall reliability.

**Cronbach Alpha**	**Standardized term based cronbach Alpha**	**Number of terms**
0.966	0.966	17

According to the overall reliability coefficient in Table 7, the standardized Cronbach's coefficient is 0.966, and the value range of the reliability coefficient is between 0 and 1. The closer the reliability coefficient is to 1, the higher the reliability will be. Therefore, the overall reliability of this questionnaire is very high.

#### Validity analysis of dimensional data in the questionnaire scale

The SPSS23 software was used to analyze the validity of the questionnaire data of the public on the current education system under the implementation of the double reduction policy, and the method of exploratory factor analysis is used to realize the test process. The specific results are shown in Table 8.

**Table 8:** Kaiser-Meyer-Olkin (KMO) and Bartlett tests.

**KMO Quantity of sampling suitability**		**0.965**
Bartlett sphericity test	The approximate chi-square	4514.523
	Degrees of freedom	136
	significance	0.000

According to the exploratory factor analysis results in Table 8, the coefficient of the Kaiser-Meyer-Olkin (KMO) test is 0.965, and the coefficient of the KMO test ranges from 0 to 1. The closer it is to 1, the better the validity of the questionnaire is. Therefore, the overall validity of this questionnaire is relatively good.

As can be seen from the significance of the spherical test in Table 8, the significance of this test is infinitely close to 0, rejecting the null hypothesis, so the questionnaire has good validity (Lv et al., 2019).

#### Difference test between dimensional data and non-dimensional data in the questionnaire

By using the SPSS23 software to analyze the difference between the non-dimensional data and the dimensional data in the questionnaire on the public's views on the current education system under the implementation of the double reduction policy, the differences of five dimensional variables in the five non-dimensional data including gender, age, identity, education background, and understanding degree of the double reduction policy were analyzed, respectively. It is concluded that under the five different demographic characteristics of gender, age, identity, education background, and understanding degree of the double reduction policy, there are differences in educational views on the equitable distribution of educational resources, the guarantee of in-school education quality, the diversification of educational training, the governance of out-of-school training, and the public education system.

In terms of gender, adopting the method of independent sample *T-*test for men and women under double reduction policy fair distribution of education resources, diversification of the quality assurance of school education, education training, training outside governance, and the public education system of the five dimensions of education view differences were analyzed, and the specific data analysis results are shown in Table 9.

**Table 9:** Analysis of gender differences in data of various dimensions.

**Variable**	**Option**	**Number of cases**	**Average Value**	**Standard Deviation**	***T* Value**	**Sig**
Dimension 1: Equitable distribution of educational resources	Male	130	20.331	4.8471	1.654	0.099
	Female	175	19.4	4.8696		
Dimension 2: Quality assurance of school education	Male	130	20.662	4.6046	1.303	0.194
	Female	175	19.937	4.9435		
Dimension 3: Diversified education and training	Male	130	15.662	3.0716	0.737	0.462
	Female	175	15.377	3.5128		
Dimension 4: Governance of out of school training	Male	130	15.569	3.3036	1.433	0.153
	Female	175	14.994	3.5791		
Dimension 5: Public education system	Male	130	16.085	3.4597	1.282	0.201
	Female	175	15.554	3.654		

According to the results of the independent sample *T*-test in Table 9, in terms of gender, the significant test values of the differences in the fair distribution of educational resources, the guarantee of school education quality, the diversification of education and training, the governance of after-school training, and the public education system are 0.099, 0.194, 0.462, 0.153, and 0.201, respectively, and these values are significantly >0.05. Therefore, it shows that there are no significant differences in the views of different genders on the five dimensions of education under the double reduction policy: the fair distribution of educational resources, the guarantee of the quality of education in school, the diversification of education and training, and the governance of out of school training and the public education system (Yang, 2022a,b).

In terms of age, identity, educational background, and understanding of the double reduction policy, the one-way ANOVA method is used to analyze the differences in educational views in the five dimensions of fair distribution of educational resources, guarantee of school education quality, diversification of education and training, governance of out of school training, and public education system under the double reduction policy. The specific analysis results are shown in Tables 10–13.

**Table 10:** Analysis of differences in age of each dimension data.

**Variable**	**Option**	**Number of cases**	**Average Value**	**Standard Deviation**	***F***	**Sig**	**Multiple comparison**
Dimension 1: Equitable distribution	<18 years old	17	20.882	4.8975	2.672	0.032	1 > 5, 2 > 5, 3 > 5, 4 > 5
of educational resources	18–28	125	19.584	4.5191			
	29–39	74	20.284	4.6802			
	40–50	59	20.542	5.2632			
	Over 50 years old	30	17.4	5.4177			
Dimension 2: Quality assurance of	<18 years old	17	20.941	4.1754	3.052	0.017	1 > 5, 2 > 5, 3 > 5, 4 > 5
school education	18–28	125	20.192	4.3491			
	29–39	74	20.919	4.9234			
	40–50	59	20.695	5.4083			
	Over 50 years old	30	17.533	4.7759			
Dimension 3: Diversified	<18 years old	17	15.941	3.6822	1.189	0.316	/
education and training	18–28	125	15.608	3.0051			
	29–39	74	15.568	3.3273			
	40–50	59	15.678	3.7621			
	Over 50 years old	30	14.267	3.4833			
Dimension 4: Governance of	<18 years old	17	16.059	3.6822	2.729	0.029	1 > 5, 2 > 5, 3 > 5, 4 > 5
out of school training	18–28	125	15.4	3.0427			
	29–39	74	15.486	3.332			
	40–50	59	15.322	4.1082			
	Over 50 years old	30	13.333	3.6135			
Dimension 5: Public	<18 years old	17	16.235	3.4009	2.263	0.062	/
education system	18–28	125	15.744	3.2376			
	29–39	74	15.851	3.61			
	40–50	59	16.475	3.5104			
	Over 50 years old	30	14.133	4.6367			

1 represents <18 years old, 2 represents 18–28 years old, 3 represents 29–39 years old, 4 represents 40–50 years old, and 5 represents more than 50 years old.

**Table 11:** Analysis of differences in investigator identity of data from various dimensions.

**Variable**	**Option**	**Number of cases**	**Average Value**	**Standard Deviation**	***F***	**Sig**	**Multiple comparison**
Dimension 1: Equitable distribution	Teachers	61	21.262	5.0922	6.924	0.000	1 > 4, 2 > 4,
of educational resources	Students	85	19.941	4.2576			3 > 4
	Parents of students	81	20.432	4.8886			
	Others	78	17.833	4.7875			
Dimension 2: Quality assurance	Teachers	61	21.902	4.9622	11.952	0.000	1 > 4, 2 > 4,
of school education	Students	85	20.435	3.8342			3 > 4
	Parents of students	81	21.235	4.6564			
	Others	78	17.718	4.8775			
Dimension 3: Diversified	Teachers	61	15.836	3.7911	2.524	0.058	/
education and training	Students	85	15.647	2.902			
	Parents of students	81	15.926	3.4525			
	Others	78	14.628	3.1504			
Dimension 4: Governance of	Teachers	61	16.131	3.8578	5.63	0.001	1 > 4, 2 > 4,
out of school training	Students	85	15.541	3.022			3 > 4
	Parents of students	81	15.506	3.3058			
	Others	78	13.936	3.4803			
Dimension 5: Public	Teachers	61	16.656	3.5397	2.563	0.055	/
education system	Students	85	15.635	3.1767			
	Parents of students	81	16	3.8665			
	Others	78	15.026	3.5964			

1 represents teachers, 2 represents students, 3 represents parents, and 4 represents others.

**Table 12:** Analysis of differences in academic qualifications of data from various dimensions.

**Variable**	**Option**	**Number of cases**	**Average Value**	**Standard Deviation**	***F***	**Sig**	**Multiple Comparison**
Dimension 1: Equitable distribution	Junior high school and below	27	16.407	5.101	6.126	0.000	1 <2, 1 <3, 1 <4
of educational resources	Senior high school	54	20.593	3.7392			
	College and undergraduate	149	20.383	4.9547			
	Master degree and above	75	19.28	4.8701			
Dimension 2: Quality assurance	Junior high school and below	27	16.444	4.4144	7.018	0.000	1 <2, 1 <3, 1 <4
of school education	Senior high school	54	21.111	3.6377			
	College and undergraduate	149	20.678	5.0927			
	Master degree and above	75	20.133	4.5332			
Dimension 3: Diversified	Junior high school and below	27	14.037	3.4023	3.141	0.026	1 <2
education and training	Senior high school	54	16.259	2.3885			
	College and undergraduate	149	15.664	3.586			
	Master degree and above	75	15.147	3.2076			
Dimension 4: Governance of out	Junior high school and below	27	12.852	3.6448	5.955	0.001	1 <2, 1 <3, 1 <4
of school training	Senior high school	54	16.019	2.9936			
	College and undergraduate	149	15.53	3.5136			
	Master degree and above	75	14.96	3.3062			
Dimension 5: Public	Junior high school and below	27	13.852	4.6962	3.890	0.009	1 <2
education system	Senior high school	54	16.426	2.8852			
	College and undergraduate	149	16.074	3.5945			
	Master degree and above	75	15.427	3.3214			

1 represents junior high school education and below, 2 represents high school education, 3 represents college and undergraduate education, and 4 represents master's degree or above.

**Table 13:** Analysis of differences in understanding of the double reduction policy among data of various dimensions.

**Variable**	**Option**	**Number of Cases**	**Average Value**	**Standard Deviation**	***F***	**Sig**	**Multiple comparison**
Dimension 1: Equitable distribution	Never heard of it	28	17.036	5.9034	9.749	0.000	1 <3, 2 <3, 1 <4, 2 <4
of educational resources	Only heard of this noun	88	18.386	4.7281			
	Have some understanding	151	20.642	4.4979			
	Very well	38	21.737	4.2279			
Dimension 2: Quality assurance	Never heard of it	28	17.286	6.3588	11.032	0.000	1 <3, 2 <3, 1 <4, 2 <4
of school education	Only heard of this noun	88	18.852	4.6942			
	Have some understanding	151	21.06	4.3837			
	Very well	38	22.421	3.4456			
Dimension 3: Diversified	Never heard of it	28	13.679	4.6671	8.841	0.000	2 <3 <4, 1 <4
education and training	Only heard of this noun	88	14.659	3.61			
	Have some understanding	151	15.94	2.8988			
	Very well	38	17.026	1.7628			
Dimension 4: Governance of	Never heard of it	28	13.107	4.7636	10.942	0.000	1 <3, 2 <3, 1 <4, 2 <4
out of school training	Only heard of this noun	88	14.216	3.813			
	Have some understanding	151	15.861	2.8309			
	Very well	38	16.711	2.4485			
Dimension 5: Public	Never heard of it	28	13.179	5.2072	13.632	0.000	1 <3, 1 <4, 2 <3 <4
education system	Only heard of this noun	88	14.841	3.7444			
	Have some understanding	151	16.298	2.9276			
	Very well	38	17.816	2.2404			

Among them, 1 stands for not having heard of it, 2 stands for having heard of it but not understanding it, 3 stands for having some understanding, and 4 stands for understanding it very much.

According to the results of the above one-way ANOVA, we can see that there are differences in educational views on the fair distribution of educational resources, the guarantee of educational quality in schools, and the governance of out-of-school training in terms of age, identity, educational background, and understanding of the double reduction policy. While the diversity of education and training and the public education system have some differences in educational views in terms of academic qualifications and understanding of the double reduction policy, there are no differences in educational views in terms of age and identity. Therefore, there are differences in educational cognitive psychology in all aspects of education under the implementation of the double reduction policy.

According to the results of correlation analysis in Table 14, under the condition of *P* <0.01, the Pearson coefficient of each dimension variable meets the requirements of significant correlation, so there is a significant correlation between each dimension variable. Therefore, it can provide the prerequisite for the existence of correlation for the construction of the structural equation model.

**Table 14:** Correlation analysis between various dimensional variables.

**Variable person** **relevance**	**Dimension 1: Equitable distribution of educational resources**	**Dimension 2: Quality assurance of school education**	**Dimension 3: Diversified education and training**	**Dimension 4: Governance of out of school training**	**Dimension 5: Public education system**
Dimension 1: Equitable distribution of educational resources	1				
Dimension 2: Quality assurance of school education	0.882**	1			
Dimension 3: Diversified education and training	0.819**	0.859**	1		
Dimension 4: Governance of out of school training	0.829**	0.880**	0.848**	1	
Dimension 5: Public education system	0.721**	0.744**	0.805**	0.798**	1

**An extremely significant correlation at the level of P <0.01.

### The structural equation model constructed based on the questionnaire data

#### Construction of structural equation model

**Figure 1:**
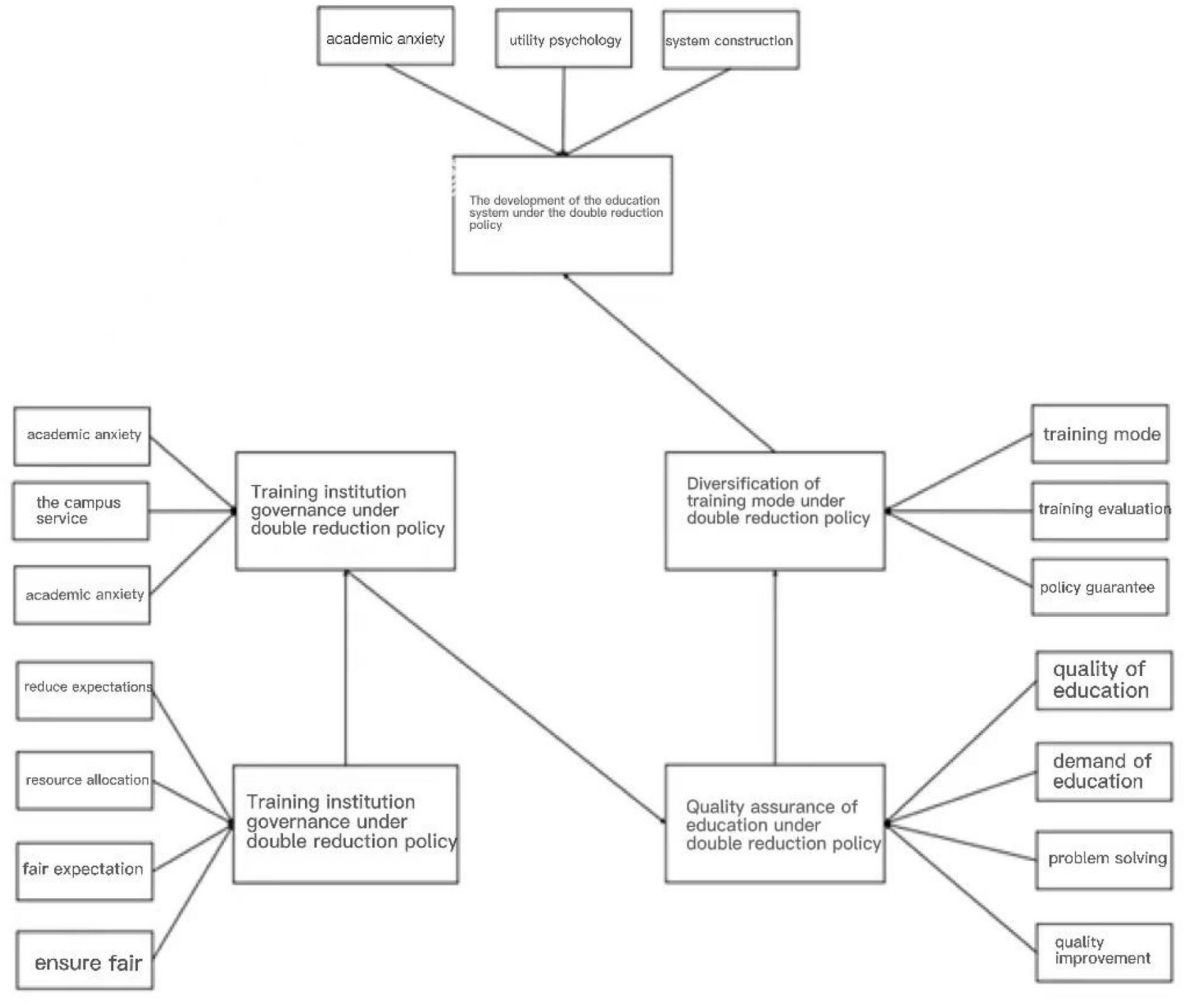
Structural equation model.

According to the method of confirmatory factor analysis, using the test model tool Amos 24 software to construct the structural equation model of the dimensional data in the questionnaire on the public's opinions on the current education system under the implementation of the double reduction policy, the path coefficient in the structural equation model through the Amos 24 software is calculated, and the influence degree of the dimensional variables of each path in the structural equation model is judged, so as to obtain the social public's psychological views on education under the implementation of the double reduction policy and provide corresponding educational psychological suggestions for the construction of later education system. The structural equation model of public opinion under the double reduction policy is shown in Figure 1.

**Figure 2:**
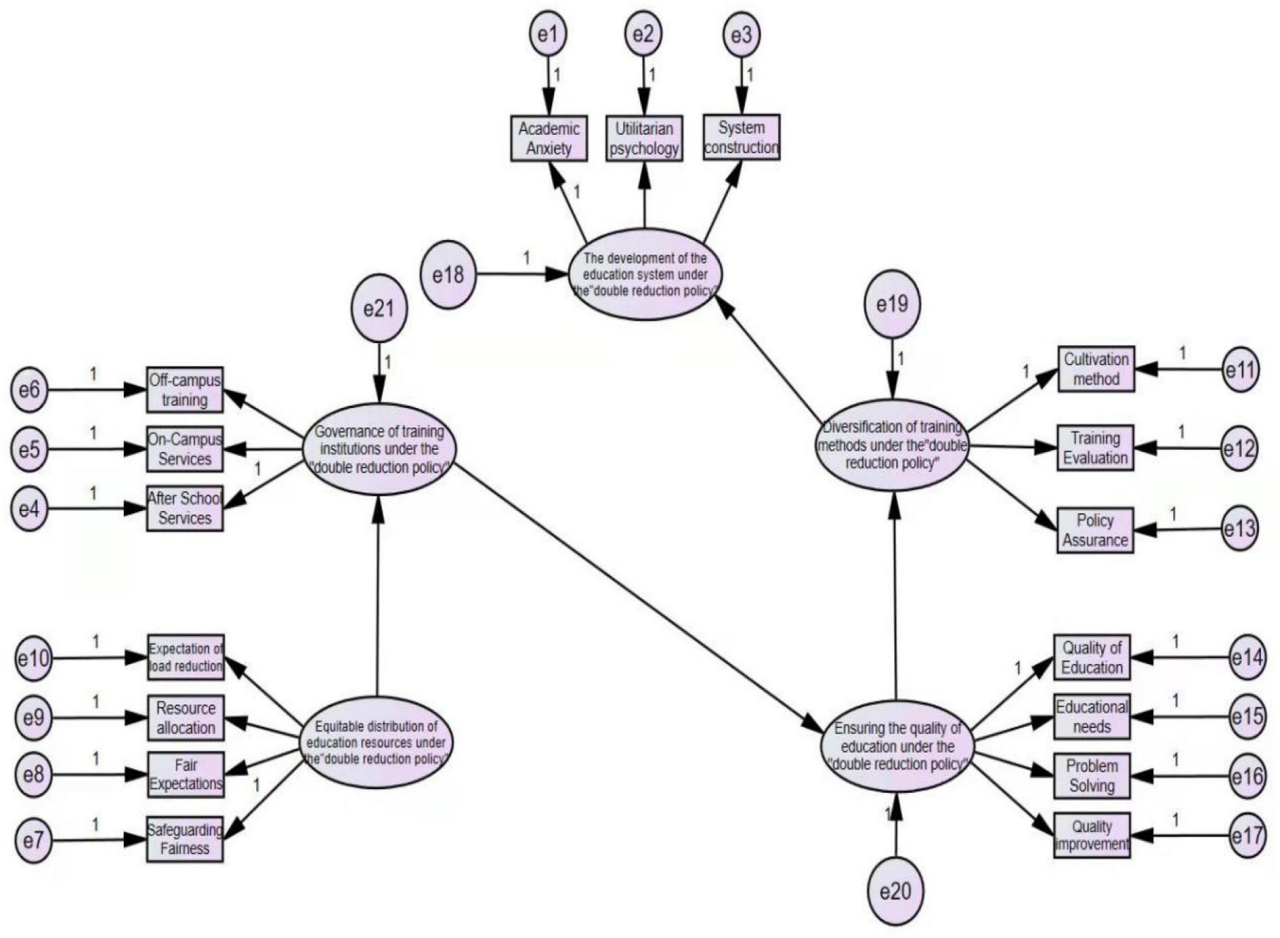
Amos structural equation model.

Then, the path coefficients of the above structural equation model are calculated using the confirmatory factor analysis method using the Amos 24 software to obtain the corresponding Amos structural equation model diagram (Chen et al., 2022). The details are shown in Figure 2.

The structural equation model calculated using the above Amos software is fitted with structural validity and convergence validity. The fitting results are shown in Tables 15, 16.

**Table 15:** Simulation fitting results of structural validity.

**Fitting index**	**Index value**	**Fitting situation**
CMIN	345.628	
DF (Degree of Freedom)	115.000	
P (Absolute Goodness-of-Fit Index/*P* value)	0.000	
CMIN/DF	3.005	<3, acceptable
GFI (Goodness of Fit Index)	0.883	> 0.8, acceptable
NFI (Normed Fit Index)	0.925	> 0.8, acceptable
CFI (Comparative Fit Index)	0.948	> 0.9, good fitting
RMSEA (Root Mean Square Error of Approximation)	0.081	<0.08, acceptable

**Table 16:** Simulation fitting results of convergence validity.

**Transverse plane**	**Index**	**Standardized load**	**Non standardized load**	**S.E**.	**C.R. (*t*-value)**	***p***	**SMC**	**C.R. (Combinatorial validity)**	**AVE (Average variance extraction)**
Development of education system under the double reduction policy	Academic anxiety	0.83	1				0.689	0.861	0.675
	Utilitarian psychology	0.909	1.068	0.056	19.042		0.826		
	System construction	0.715	0.797	0.058	13.754		0.511		
Governance of training institutions under the double reduction policy	After class service	0.819	1				0.671	0.838	0.633
	On campus services	0.799	1.151	0.068	16.819		0.638		
	Off campus training	0.768	1.088	0.069	15.862		0.59		
Fair distribution of educational resources under the double reduction policy	Guarantee fairness	0.838	1				0.702	0.881	0.649
	Fair expectation	0.818	0.905	0.052	17.393		0.669		
	Resource allocation	0.822	0.977	0.055	17.727		0.676		
	Burden reduction expectation	0.74	0.781	0.052	14.993		0.548		
Diversified training methods under the double reduction policy	Training mode	0.83	1				0.689	0.857	0.667
	Cultivation Evaluation	0.826	1.155	0.066	17.476		0.682		
	Policy guarantee	0.794	0.971	0.058	16.642		0.63		
Guarantee of education quality under the double reduction policy	Quality of Education	0.792	1				0.627	0.889	0.667
	Educational needs	0.811	1.007	0.062	16.176		0.658		
	Problem solving	0.809	0.924	0.057	16.213		0.654		
	Quality improvement	0.854	0.962	0.055	17.397		0.729		

Based on confirmatory factor analysis, this study uses the Amos software to construct a structural equation model of the public's view on the education system under the implementation of the double reduction policy. According to Table 15, the fitting value of CMIN/DF is 3.005. It is very close to 3, and the adaptation is relatively ideal. The fitting value of RMSEA is 0.081, which is very close to 0.08, and the fitting is relatively ideal. The fitting values of GFI and NFI are 0.883 and 0.925, respectively, which are >0.8, and the results are well matched. The fitting value of CFI is 0.948, >0.9, and the results are well adapted. Therefore, on the whole, the structural equation model of educational cognitive psychology of the public under the implementation of the double reduction policy is well adapted (Wan and Cheng, 2021).

This study analyzes the convergence validity of the data from five dimensions, namely, the development of the education system, the governance of training institutions, the fair distribution of educational resources, the diversification of training methods, and the assurance of educational quality. First, the standardized compliance of each index in the five dimensional data is between 0.7 and 0.9. Second, the combined validity CR values of the five dimensional data are |>0.7, which means that the higher the consistency of internal data, the more convergent. The average variance extraction volume AVE value of the last five dimensions of data is >0.5, which indicates that the potential variables of the five dimensions of data have an average explanatory ability to their corresponding potential variables, so the convergence validity is good. Therefore, the data on the five dimensions of the development of the education system, the governance of training institutions, the fair distribution of educational resources, the diversification of training methods, and the assurance of educational quality under the double reduction policy have convergent validity, so it can be used as a psychological research model for the views of the public on education under the double reduction policy. At the same time, compared with the previous research results on the double reduction policy, using the SPSS software and Amos software to analyze the data in the questionnaire on the public's views on the current education system under the double reduction policy, it is more prominent that this research is based on quantitative analysis. The method is used to explore the psychological cognition of the public about the current education system under the implementation of the double reduction policy.

## Research on public psychology based on the results of data analysis

According to the above research results, under the implementation of the double reduction policy, the development of the education system, the governance of training institutions, the fair distribution of educational resources, the diversification of training methods, and the quality assurance of education are the five dimensions of data and the analysis of demographic variables, and the group characteristics of the public collected in this questionnaire are mainly those aged between 29 and 39 years who have received higher education. Female parents who have a certain understanding of the double reduction policy and their views on all aspects of education under the implementation of the double reduction policy are relatively unified, indicating that men and women have the same cognitive psychology of future education under the influence of the double reduction policy.

In terms of the dimensions of fair distribution of education, the guarantee of the quality of in-school education, and the governance of out-of-school training, those over the age of 50 years who have not received higher education do not know much about the double reduction policy and are not the social masses of parents and teachers in their own identity. There are obvious differences between the view of fair distribution of education and that of other mass groups. Finally, on the dimension of educational diversity and public education system, there are obvious psychological differences in educational views among the public who have not received high school education, have received high school education, and have a different understanding of the double reduction policy.

Through the structural equation model of the public's mentality of the current education system under the implementation of the double reduction policy constructed by the quantitative analysis research method, we can see that the public's ultimate psychological vision for domestic education is to achieve fair development of China's public education system, and the public psychological point of view is that through the management of educational courses of training institutions, it can have a good role in promoting the fair distribution of educational resources in China. To achieve the goal of diversifying education and training methods, the fair and just development of the domestic public education system is ultimately ensured.

## Conclusion

This study collects data on the public's psychological views on education under the double reduction policy by means of questionnaires and conducts variance analysis and structural equation model tests on the collected data. Education, the public who do not know much about the double reduction policy, and their identity are not parents and teachers. Under the double reduction policy, the public is treated with fair distribution of education, on-campus education quality, off-campus training governance, education and training diversity, and the public education system. These five aspects have different psychological views from other members of the public, and the general psychological view of the public on education is to rectify the education and training industry through the implementation of the “double reduction policy,” in order to ensure the fair distribution of educational resources, to ensure the quality of school education, so as to realize the diversification of education and training methods to ensure the fair and just development of the domestic public education system. Therefore, based on the above-mentioned public opinion on education psychology under the double reduction policy, the following policy suggestions are put forward:

(1) To ensure the balanced development of the domestic education system, it is necessary to popularize the ideological connotation of the double reduction policy to the public, so as to ensure that the older and uneducated social public's lack of understanding of the implementation of the policy can be improved, so as to ensure the public's perception of the education system.
(2) To ensure the balanced development of the domestic education system, it is necessary to rectify the excessive supplementary courses in the education and training industry, so as to ensure the fair distribution of domestic educational resources and the improvement of school education quality and build a fair education system with diversified training methods.

At the same time, this research analyzes the psychological cognition of the public about the current education system under the implementation of the double reduction policy by means of quantitative analysis, so as to provide corresponding insights for the public's psychological cognition research on the current education system under the implementation of the double reduction policy.

## Data availability statement

The original contributions presented in the study are included in the article/supplementary material, further inquiries can be directed to the corresponding author/s.

## Author contributions

XZ wrote the paper, WZ built the model, and KZ revised the paper. All authors contributed to the article and approved the submitted version.

## Conflict of interest

The authors declare that the research was conducted in the absence of any commercial or financial relationships that could be construed as a potential conflict of interest.

## Publisher's note

All claims expressed in this article are solely those of the authors and do not necessarily represent those of their affiliated organizations, or those of the publisher, the editors and the reviewers. Any product that may be evaluated in this article, or claim that may be made by its manufacturer, is not guaranteed or endorsed by the publisher.
